# Genomic analysis of the meningococcal ST-4821 complex–Western clade, potential sexual transmission and predicted antibiotic susceptibility and vaccine coverage

**DOI:** 10.1371/journal.pone.0243426

**Published:** 2020-12-10

**Authors:** Jay Lucidarme, Bingqing Zhu, Li Xu, Xilian Bai, Yuan Gao, Juan José González-López, Robert Mulhall, Kevin J. Scott, Andrew Smith, Paola Stefanelli, Bianca Stenmark, Paul Torpiano, Georgina Tzanakaki, Ray Borrow, Zhujun Shao

**Affiliations:** 1 Meningococcal Reference Unit, Public Health England, Manchester Royal Infirmary, Manchester, United Kingdom; 2 National Institute for Communicable Disease Control and Prevention, Chinese Center for Disease Control and Prevention, Beijing, PR China; 3 Department of Clinical Microbiology, Hospital Universitari Vall d’Hebron, Barcelona, Spain; 4 Irish Meningitis and Sepsis Reference Laboratory, Children’s Health Ireland at Temple Street, Dublin, Ireland; 5 Bacterial Respiratory Infection Service, Scottish Microbiology Reference Laboratory, Glasgow Royal Infirmary, Glasgow, United Kingdom; 6 College of Medical, Veterinary & Life Sciences, Glasgow Dental Hospital & School, University of Glasgow, Glasgow, United Kingdom; 7 Department of Infectious Diseases, Istituto Superiore di Sanità, Rome, Italy; 8 Department of Laboratory Medicine, Faculty of Medicine and Health, Örebro University, Örebro, Sweden; 9 Department of Child and Adolescent Health, Mater Dei Hospital, Msida, Malta; 10 National Meningitis Reference Laboratory, Dept. of Public Health Policy, School of Public Health, University of West Attica, Athens, Greece; Emory University School of Medicine, UNITED STATES

## Abstract

**Introduction:**

The ST-4821 complex (cc4821) is a leading cause of serogroup C and serogroup B invasive meningococcal disease in China where diverse strains in two phylogenetic groups (groups 1 and 2) have acquired fluoroquinolone resistance. cc4821 was recently prevalent among carriage isolates in men who have sex with men in New York City (USA). Genome-level population studies have thus far been limited to Chinese isolates. The aim of the present study was to build upon these with an extended panel of international cc4821 isolates.

**Methods:**

Genomes of isolates from Asia (1972 to 2017), Europe (2011 to 2018), North America (2007), and South America (2014) were sequenced or obtained from the PubMLST *Neisseria* database. Core genome comparisons were performed in PubMLST.

**Results:**

Four lineages were identified. Western isolates formed a distinct, mainly serogroup B sublineage with alleles associated with fluoroquinolone susceptibility (MIC <0.03 mg/L) and reduced penicillin susceptibility (MIC 0.094 to 1 mg/L). A third of these were from anogenital sites in men who have sex with men and had unique denitrification gene alleles. Generally 4CMenB vaccine strain coverage was reliant on strain-specific NHBA peptides.

**Discussion:**

The previously identified cc4821 group 2 was resolved into three separate lineages. Clustering of western isolates was surprising given the overall diversity of cc4821. Possible association of this cluster with the anogenital niche is worthy of monitoring given concerns surrounding antibiotic resistance and potential subcapsular vaccine escape.

## Introduction

*Neisseria meningitidis*, the meningococcus, is a leading cause of meningitis and sepsis. Six serogroups (A, B, C, W, X and Y) cause the majority of invasive meningococcal disease (IMD) globally. Prior to 2003/4, meningococcal serogroup A (MenA) was the most common cause of IMD in China with periodic epidemics [1]. In 1982 the country introduced MenA polysaccharide vaccination after which IMD stabilised at 0.2 to 1.0 per 100,000 [2].

In 2003/4 and 2004/5, a number of serogroup C (MenC) outbreaks occurred in Anhui province due to a novel strain—the Sequence Type (ST)-4821 complex (cc4821) [2]. A national survey of case, case-contact, and carrier isolates in 2004/5 revealed the existence of MenC cc4821 in disease and/or carriage in 11 other provinces [2]. Subsequent analysis of geo-temporally diverse Chinese MenC isolates identified several cc4821 case and carrier isolates dating back as far as 1978 [3]. In 2005 the serogroup A polysaccharide vaccine was replaced with a MenA and MenC polysaccharide vaccine [4]. In recent years MenC cc4821 has become a leading and widespread cause of IMD in China. cc4821 has also emerged as a leading cause of serogroup B (MenB) disease which has tended to be more sporadic in nature [4]. cc4821 isolates express diverse PorA subtypes, however, the majority of the MenC cases, particularly those associated with the outbreaks, possessed PorA P1.7–2,14 [4].

High-resolution phylogenetic analyses of small numbers (n = 22 to 32) of Chinese cc4821 case and carrier isolates have found that cc4821 comprises two distinct groups—group 1 (or group I) and group 2 (or group II) [5, 6]. The group 1 isolates were very closely related to one-another and included a subcluster of isolates with PorA P1.7–2,14 (the epidemic clone [6]). The group 2 isolates were relatively diverse and had a lower proportion of invasive isolates. In the study by Guo *et al*. (2018) [6] the most common DNA gyrase subunit A (*gyrA*) allele among the group 2 isolates, as well as the majority (22/29) of a distinct panel of group 2 isolates from Shanghai, was a wild type allele, allele 12, which is associated with susceptibility (MIC ≤0.03 mg/L; EUCAST v10.0) to ciprofloxacin among 137/138 PubMLST *Neisseria* isolates (accessed 10^th^ September 2020). Ciprofloxacin is commonly used for prophylaxis against IMD. The remaining group 2 isolates possessed a variety of *gyrA* alleles with resistance-associated mutations (MICS >0.03 mg/L; EUCAST v10.0) that affect amino acid residue 91 [7]. All of the group 1 isolates possessed *gyrA* allele 71, containing the resistance-associated mutation T91I.

Concerns about antibiotic resistance are confounded by the existence of multiple MenB strains in both groups 1 and 2 [5, 6] as a result of multiple capsular switch events [6]. No MenB vaccine is currently licensed in China. Two subcapsular vaccines that are licensed elsewhere afford strain coverage dependant on the presence and expression of cross-reactive peptide variants among the prevailing meningococcal population [8, 9]. The 4CMenB vaccine potentially covers isolates sufficiently expressing PorA P1.4, and/or a sufficiently cross-reactive factor H-binding protein (fHbp) variant 1 peptide, and/or a Neisseria adhesin A (NadA) variant NadA-1 or NadA-2/3 peptide, and/or a cross-reactive neisserial heparin–binding antigen (NHBA) peptide. Coverage by MenB-fHbp is dependent on sufficient expression of fHbp. Potential coverage of individual isolates is determined using the ELISA-based Meningococcal Antigen Typing System (MATS; 4CMenB) [10] or the flow-cytometry based Meningococcal Antigen Surface Expression (MeASurE; MenB-fHbp) [11] assays. A genetic version of MATS (gMATS) has also been developed based on a large international MATS dataset [12]. The emergence of a serogroup W cc4821 strain in both carriage and disease is also a cause for concern [13], although highly effective serogroup W conjugate vaccines are available.

Although invasive disease due to cc4821 is rare outside of Asia, Guo *et al*. (2018) previously noted the expansion of non-Chinese cc4821 isolates within the PubMLST *Neisseria* database [6]. In a recent meningococcal carriage study in 706 men who have sex with men (MSM) in New York City (USA), cc4821 was the most prevalent clonal complex identified accounting for 14.3% (24/168) isolates [14]. Members of another strain, ST-11 complex lineage 11.2, have caused multiple outbreaks of IMD in MSM in Europe and the USA and a large multistate outbreak of urethritis among predominantly heterosexual men in the USA [15]. The latter strain (US_NmUC) has recently been detected in proctitis in MSM in the United Kingdom [16]. The strains responsible have shown several adaptations to the anogenital niche including the acquisition of active/efficient denitrification genes (*aniA* and *norB*) to enable growth in an anaerobic environment [17, 18]. A recent study in gonococci found an association between loss of function (LOF) mutation in the efflux pump component, MtrC, and cervical infection. This extended to US_NmUC in which 8.7% of isolates possessed LOF mutations versus 0.62% in a large invasive disease collection [19].

In the present study, we expanded upon earlier work by sequencing additional Chinese genomes and performing high resolution phylogenetic analyses on these and an extended panel of all cc4821 genomes from multiple countries on several continents that are currently available on the PubMLST Neisseria database (accessed 12^th^ September 2019). We also charted the distribution of ciprofloxacin resistance-associated *gyrA* mutations and *penA* mutations associated with reduced susceptibility to penicillin; the nitrite reductase gene *aniA*; and potential cc4821 strain coverage by subcapsular (MenB) vaccines. Predicted functionality of MtrC was assessed in a set of anogenital isolates.

## Methods

### Genome sequencing and database searches

New genome sequences were obtained for 138 Chinese cc4821 isolates collected between 1978 to 2017, inclusive (S1 Table). DNA was extracted from overnight cultures using the Wizard genomic DNA purification kit (Promega, WI, USA) according to the manufacturer’s instructions. Paired end libraries were generated with average insert lengths of 350 bp or 500 bp. Sequencing was performed on Illumina HiSeq 2000, 2500 or 4000 platforms (Illumina, CA, USA).Genome assembly was performed using SOAPdenovo (release 1.04; http://soap.genomics.org.cn/soapdenovo.html).

Other cc4821 genomes were obtained from the PubMLST *Neisseria* isolate database (https://pubmlst.org/neisseria/) using the ‘search or browse database’ function (accessed 12/09/2019). Isolates with >4 ST-4821 alleles among clonal complex-unassigned isolates (incomplete MLST profiles or unassigned STs) were also included.

Associated metadata and genotypic data were exported using the ‘export dataset’ function. Stated capsular groups are from the ‘capsule_group’ field which is determined automatically from the contents of the serogroup and/or genogroup fields in PubMLST.

### Phylogenetic analyses and identification of putative recombination events

Genomes were compared using the PubMLST genome comparator tool [20] in conjunction with the meningococcal core genome MLST scheme (*N*. *meningitidis* cgMLST v1.0) [21] using an ST-41/44 complex genome (PubMLST ID 19266; M01-240149) as an outgroup. The resulting distance matrixes were visualised and annotated using SplitsTree4 (version 4.12.8) [22].

Putative recombinations were identified by comparing genomes in term of all NEIS loci and identifying blocks of differing genes between closely related isolates.

### BLAST searches and nucleotide/amino acid sequence alignments

Allelic sequence data were obtained from the PubMLST *Neisseria* database using the ‘sequence attribute search’ tool. BLAST searches were performed using the PubMLST BLAST tool. Nucleotide and amino acid sequence alignments were performed using BioEdit (version 7.0.9.0) [23].

### Classification of antibiotic susceptibility

Minimum inhibitory concentration (MIC) breakpoints for antibiotic sensitivities differ according to publication and by the organisations that define them, including occasional revisions over time. Current EuCAST (v10.0) breakpoints for intravenous administration of penicillin are susceptible ≤0.06 mg/L and resistant >0.25 mg/L. Studies in animal models, however, suggest an upper breakpoint of >1 mg/L for resistance [24]. Current EuCAST (v10.0) breakpoints for ciprofloxacin for use in the prophylaxis of meningococcal disease are susceptible ≤0.03 mg/L and resistant >0.03 mg/L.

For the present study, isolates possessing up to five mutations (F504L, A510V, I515V, H541N and I566V) within the deduced amino acid sequence for a 402 bp fragment (*penA*) of the penicillin binding protein 2 gene (PubMLST locus NEIS1753) were predicted to have reduced susceptibility to penicillin (0.094 to 1 mg/L) in accordance with Taha *et al*. (2007) [25]. Isolates with alterations to residues T91 or D95 of the deduced amino acid sequence of the DNA gyrase gene (*gyrA*; PubMLST locus NEIS1320) were predicted to be resistant (MIC, ≥0.03 mg/L) to ciprofloxacin in accordance with Hong *et al*., 2013 [7]. Alterations to residues D86, S87, S88 or E91 of the deduced amino acid sequence of the DNA topoisomerase IV subunit A gene (parC; PubmLST locus NEIS1525) were also noted having previously been associated with incrementally higher ciprofloxacin MICs when observed in tandem with *gyrA* mutations in *N*. *gonorrhoeae* [26].

## Results

### Final genome panel

In addition to the 138 new genomes, the database search yielded 50 genomes including 49 cc4821 genomes and a single ST-unassigned genome (PubMLST ID 85386) that had four MLST alleles in common with both ST-4821 and ST-8. The latter genome was subsequently assigned to ST-14840 that, in accordance with the rules by which 7-locus STs are assigned (https://pubmlst.org/neisseria/info/complexes.shtml), was assigned to the ST-8 complex. Given the uncertainty regarding the isolate’s true descent it was included in subsequent analyses employing high resolution cgMLST. Collectively, the genomes were from carriage (n = 121), invasive disease (n = 57), anogenital sites (n = 6) and unspecified (n = 4). The isolates were isolated between 1972 and 2018 in Asia (1972 to 2017, including China, n = 170; Japan, n = 1); Europe (2011 to 2018, including UK, n = 6; Italy, n = 3; Ireland, n = 2; Greece, n = 1; Malta, n = 1; Spain. N = 1; Sweden, n = 1); North America (2007, USA, n = 1); and South America (2014, Brazil, n = 1) (**Table 1**).

**Table 1:** Geo-temporal origins of genomes used in the study.

Continent	Country	Disease state								Total
		Carrier		Invasive		Urogenital		Unspecified		
		n	Years	n	Years	n	years	n	years	
Asia	China	112	1972–2017	54	1985–2017			4	1980–2005	170
	Japan			1	2017					1
Europe	UK			1	2014	5	2011–2018			6
	Greece	1	2018							1
	Malta	1	2018							1
	Italy	2	2016	1	2017					3
	Ireland	2	2017							2
	Spain					1	2018			1
	Sweden	1	2018							1
North America	USA	1	2007							1
South America	Brazil	1	2014							1
Total		121		57		6		4		188

### Population structure

**Fig 1:**
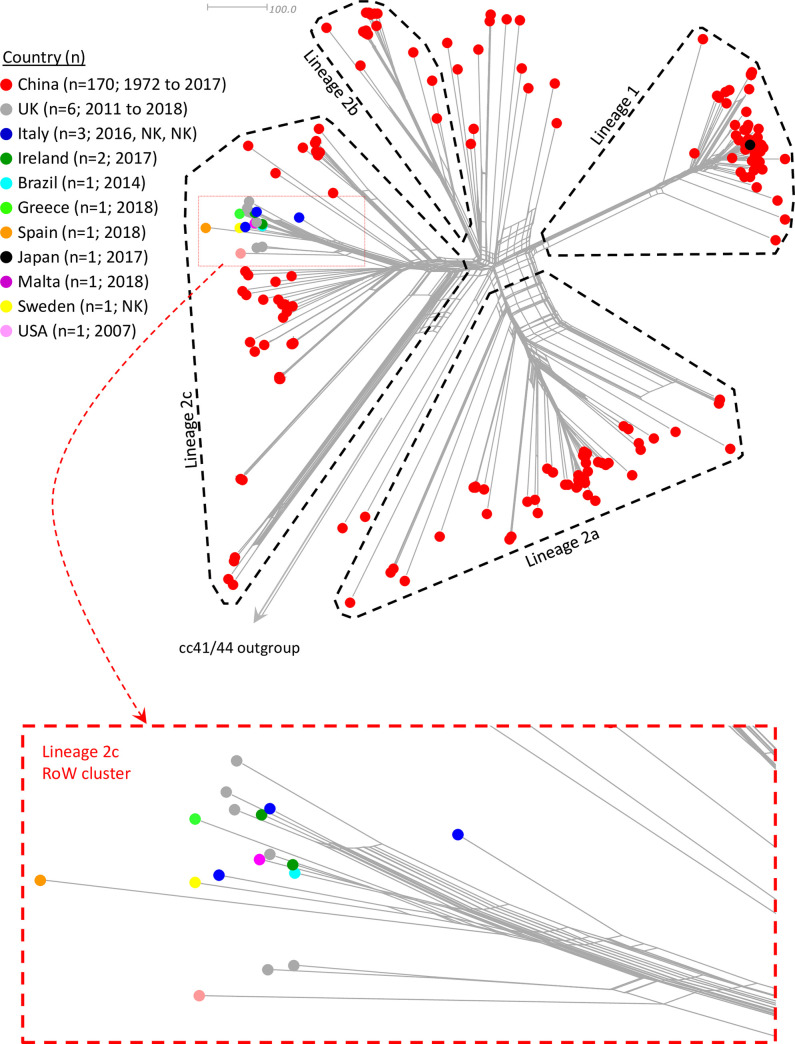
Population structure of geo-temporally diverse cc4821 isolates highlighting geographic origins. With the exception of a Japanese isolate in lineage 1, all of the non-Chinese isolates belonged to a discreet ‘Rest of the world’ (Row) cluster. The phylogeny was based on a core genome (1605 loci) comparison. The scale bar represents the number of different loci. UK = United Kingdom.

**Fig 2:**
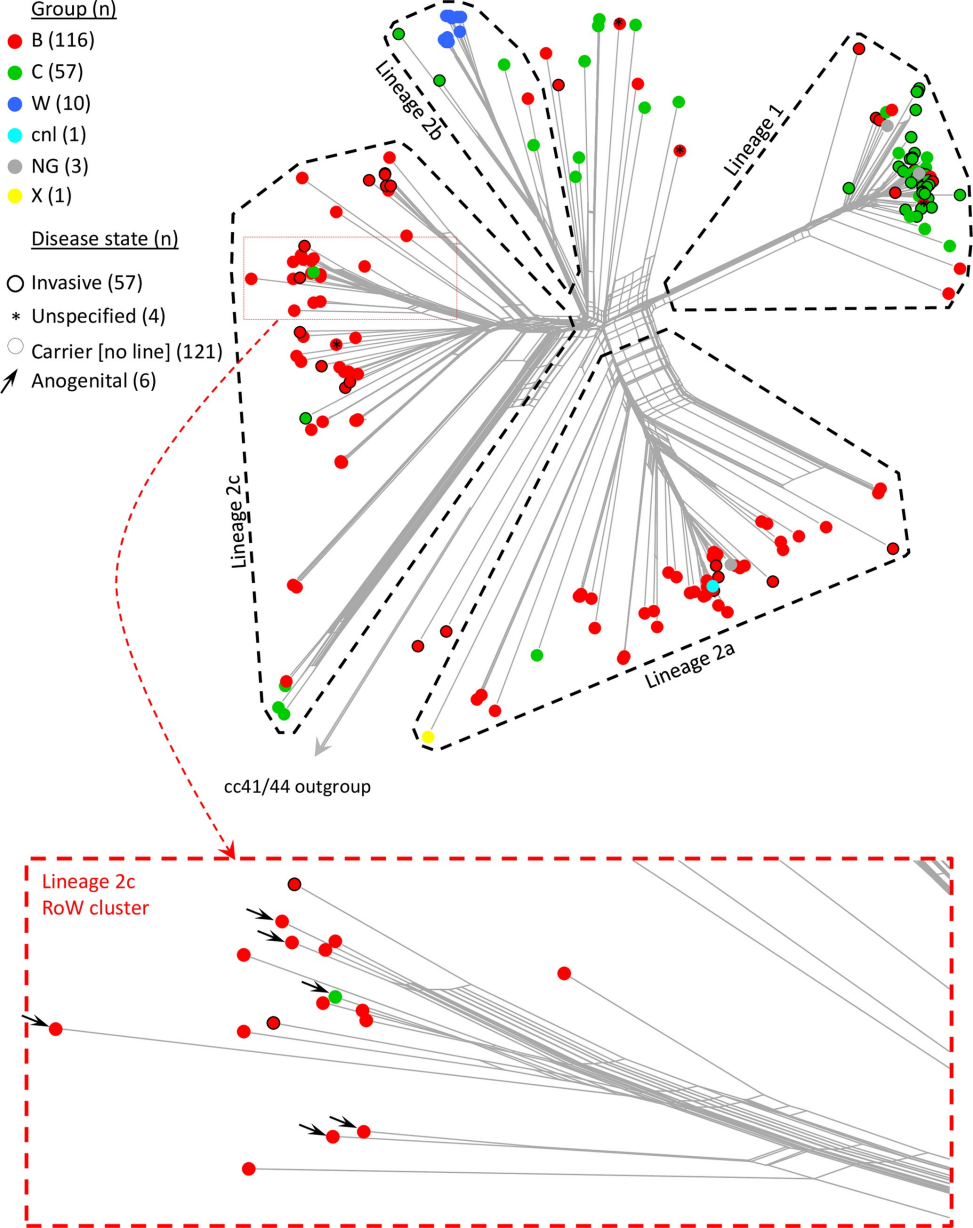
Population structure of geo-temporally diverse cc4821 isolates highlighting serogroup and disease status. Group B and C isolates are interspersed throughout though lineage 1 is predominantly MenC while lineages 2a and 2c are mainly MenB. The MenW isolates formed a discreet cluster in lineage 2b. cnl = capsule null. NG = non-groupable.

**Figs 1 and 2** show the population structure of the genomes highlighting geographical origins, and serogroup and disease status, respectively. The cc4821 isolates were diverse and formed four distinct lineages among several relatively diffuse isolates. To maintain a linkage to the previous studies by Zhu *et al*. (2015) [5] and Guo *et al*. (2018) [6] these were designated lineage 1 (corresponding to Zhu/Guo group I/1) and lineages 2a, 2b and 2c (collectively corresponding to Zhu/Guo group II/2). The positions of isolates used in the earlier studies are shown in **S1 Fig**. The ST-14840 (cc8) isolate, a Spanish urethral swab isolate from 2018, belonged to lineage 2c confirming it was descended from cc4821 meningococci, rather than those of cc8 to which it is officially assigned. Each of the four lineages was represented by isolates from multiple Chinese provinces **(S2 Fig)**. With the exception of a single diffuse isolate from China in 1980, PorA P1.7–2,14 was only observed within a discrete lineage 1 cluster that included the epidemic clone isolates as designated by Guo *et al*. (2018) [6] **(S3 Fig)**. There were 55 sequence types among the study genomes. Among the predominant sequence types, ST-4821 was distributed between lineage 1 and several diffuse isolates from 1973 to 1985 (n = 5) and 2009 (n = 1). ST-5664 and ST-9454 were confined to lineage 2a. ST-8491 was confined to MenW isolates of lineage 2b. ST-3200 and ST-5798 were confined to lineage 2c **(S4 Fig)**.

The geo-temporal composition and disease status of the four lineages was as follows:

#### Lineage 1

The majority of lineage 1 isolates (n = 51/52, 2004 to 2017) were from China including 16 carrier isolates belonging to MenC (n = 10; 2005 to 2014), MenB (n = 4; 2009–2016), and serogroup unknown (n = 2; 2007 and 2009); 34 invasive isolates belonging to MenB (n = 6, 2008 to 2014) and MenC (n = 28; 2004 to 2016); and a single MenB isolate with disease status unknown (2005). The remaining isolate was an invasive MenC isolate from Japan (2017).

#### Lineage 2a

All lineage 2a isolates (n = 49; 2005 to 2017) were from China and most (n = 45/49) were MenB. The lineage included 44 carrier isolates (2005 to 2016; MenB, n = 40; MenC, n = 1; MenX, n = 1; capsule null (cnl), n = 1; and unknown, n = 1), and five invasive isolates (2011 to 2017; all MenB) **(Fig 2)**. Four of the invasive isolates belonged to a distinct cluster of relatively closely related isolates.

#### Lineage 2b

All of the lineage 2b isolates (n = 15; 1972 to 2017) were from China. The majority (n = 13/15) were carrier isolates including a single MenB (1980), two MenC (1972 and 2007), and ten MenW isolates (2009 to 2017). The two invasive isolates (2002 and 2005) belonged to MenC and formed a distinct sublineage.

#### Lineage 2c

The majority (n = 40/57) of the lineage 2c isolates were from China including 29 carrier isolates belonging to MenB (n = 26; 1977 to 2016) and MenC (n = 3; 2016 to 2017), ten invasive isolates belonging to MenB (n = 9; 2005 to 2013) and MenC (n = 1; 2005) **(Fig 2)**, and a single MenB isolate (2005) with unknown disease status.

The remaining lineage 2c isolates (n = 17/57) were from nine countries in Europe and North and South America and formed a distinct cluster within lineage 2c designated the ‘cc4821 lineage 2c RoW (rest of world) cluster’ **(Fig 1)**. These included carrier MenB isolates from meningococcal carriage studies in the USA (n = 1 isolate; 2007; high school children) [27]; Brazil (n = 1 isolate; 2014; adolescents) [28]; Italy (n = 2 isolates; 2016; young adults); Ireland (n = 2 isolates; 2017; Universities); Greece (n = 1 isolate; 2018; adolescents), Sweden (n = 1 isolate; 2018; universities) and Malta (n = 1 isolate; 2018; university); and invasive MenB isolates from Italy (n = 1; 2017) and the UK (n = 1; 2014) **(Fig 2)**. The remaining six lineage 2c isolates were all from anogenital sites among men who have sex with men (MSM). These included three UK MenB isolates (2011, 2012 and 2013) that were incidentally isolated from rectal swabs during routine gonococcal screening [29]. Two of these were from dual meningococcal/gonococcal coinfections. A further UK MenB rectal swab isolate (2017; age 20 to 30 years) was from a meningococcal/gonococcal coinfected asymptomatic contact of a gonococcal urethritis patient. Another MenB isolate was from a urethral swab from a meningococcal/*Chlamydia trachomatis* coinfected sex worker with symptoms of urethritis in Spain (2018; 20 to 30 years). The final isolate belonged to MenC and was a urethral swab isolate from a UK bisexual male practicing protected insertive sex and displaying symptoms of urethritis (2018; 30 to 40 years). Coinfections were ruled out for *C*. *trachomatis*, *Mycoplasma genitalium* and gonococcus.

#### Diffuse isolates

The diffuse isolates that were not assigned to particular lineages (n = 15; 1973 to 2011) were all from China and comprised ten carrier isolates including MenB (n = 2; 2009 to 2011) and MenC (n = 8; 1973 to 2008) isolates; three invasive MenB isolates (1985 to 2011); and two MenB isolates with unspecified disease status (1980 to 1985).

### Antibiotic resistance determinant genotyping

**Fig 3:**
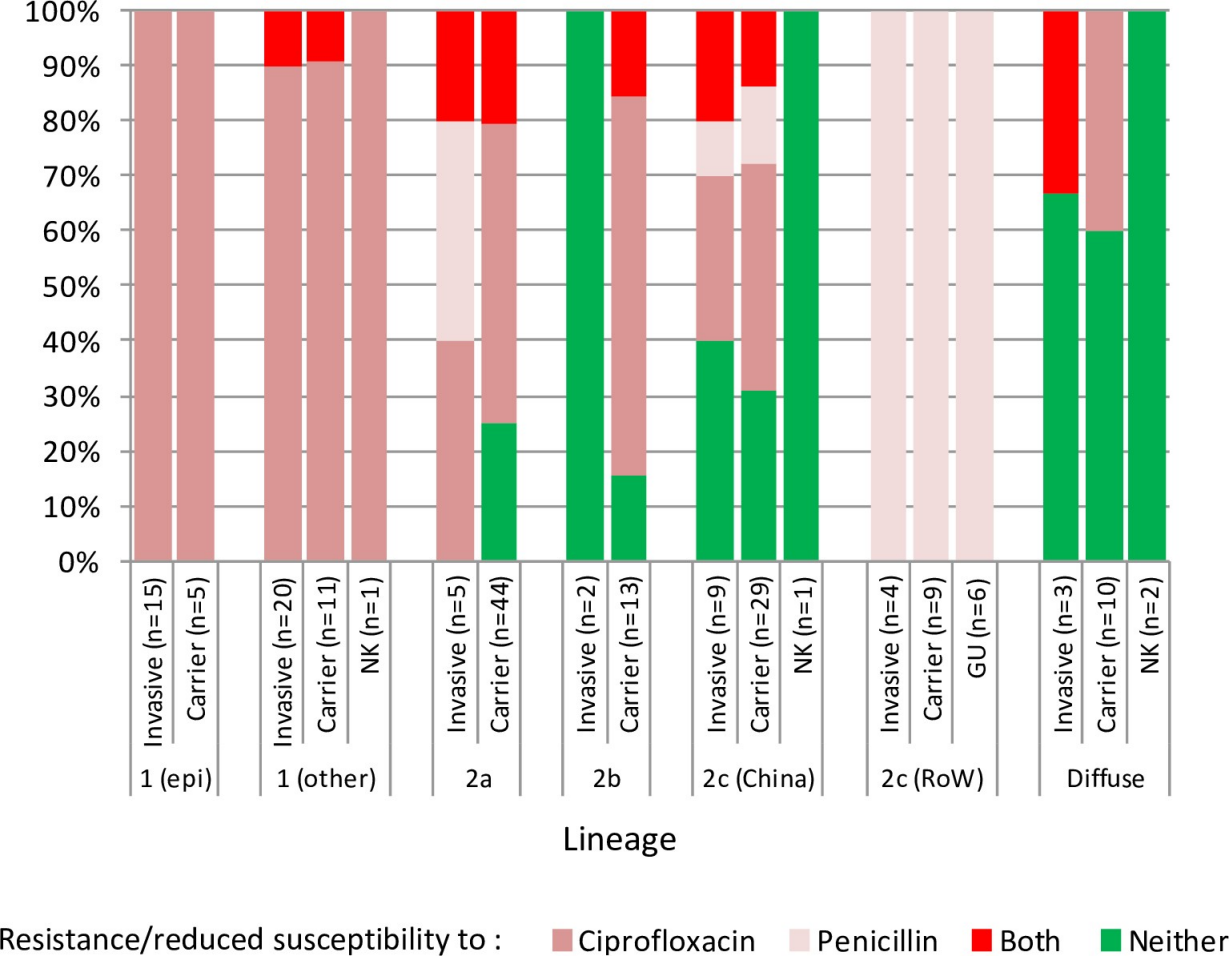
cc4821 lineage/sublineage distribution of fluoroquinolone resistance-associated *gyrA* alleles and *penA* alleles associated with reduced penicillin susceptibility. *gyrA* alleles were considered to be associated with fluoroquinolone resistance (MIC ≥ 0.03 mg/L) if they possessed mutations affecting residues T91 or D95 of the corresponding peptide. *penA* alleles were considered to be associated with reduced penicillin susceptibility (0.094 to 1 mg/L) if they encoded the following mutations: F504L, A510V, I515V, H541N and I566V.

The different lineages/sublineages contained varying distributions of isolates with fluoroquinolone resistance-associated (MICs ≥ 0.03 mg/L) *gyrA* and *parC* alleles and *penA* alleles associated with reduced penicillin susceptibility (0.094 to 1 mg/L) **(Fig 3)**. Detailed lineage breakdowns of genotypic antibiotic resistance determinants are depicted in **S5 Fig** and as follows:

#### Lineage 1

All 52 of the lineage 1 isolates possessed mutant (resistance-associated) *gyrA* alleles (51x allele 71 and 1x allele 326). Among these, 45 isolates (including 18/20 epidemic clone isolates) possessed wild type (WT; susceptibility-associated) *penA* alleles. One of these, a MenB carrier isolate (2009), also possessed a mutant *parC* allele (mutation S88P). Three isolates, including an invasive MenB (2014) and MenC (2012) isolate and a carrier MenB isolate (2016), possessed mutant *penA* alleles (5x reduced susceptibility-associated alleles). A further three carrier isolates (including 2/20 epidemic clone) and one invasive isolate did not have full length *penA* sequence data. The single Japanese epidemic clone isolate possessed mutant *gyrA* and *parC* (mutation S87I) alleles and WT *penA* alleles.

#### Lineage 2a

Among the lineage 2a carrier isolates, 8/40 MenB isolates (2011 to 2016) and 1/1 MenX isolates (2009) possessed mutant *gyrA* and *penA* alleles. Of these, two MenB isolates (2014 to 2016) also possessed mutant *parC* alleles (mutation E91K). A further 22/40 MenB isolates (2006 to 2016), and a single cnl (2009) and serogroup-unknown (2016) isolates possessed mutant *gyrA* and WT *penA* alleles. Of these, a single MenB isolate (2013) also possessed a mutant *parC* allele (mutation S87I). The remaining 10/40 MenB (2005 to 2014) and 1/1 MenC (2005) carrier isolates possessed WT *gyrA* and *penA* alleles.

Among the five MenB lineage 2a invasive isolates, one (2017) possessed mutant *gyrA* and *penA* alleles, two (2011 to 2013) possessed mutant *gyrA* alleles and WT *penA* alleles, and two (both 2014) possessed WT *gyrA* and mutant *penA* alleles.

#### Lineage 2b

Among the lineage 2b carrier isolates, 1/1 MenB (1980) and 1/2 MenC (1972) possessed WT *gyrA* and *penA* alleles. The other MenC isolate (2007) and 8/10 MenW isolates (2009 to 2017) possessed mutant *gyrA* and WT *penA* alleles. The remaining 2/10 (2014 and 2016) MenW isolates possessed mutant *gyrA* and *penA* alleles. The two invasive MenC isolates (2002 and 2005) possessed WT *gyrA* and *penA* alleles.

#### Lineage 2c

Among the Chinese lineage 2c MenB carrier isolates, 9/26 isolates (1977 to 2011) possessed WT *gyrA* and *penA* alleles, 12/26 (2005 to 2016) possessed mutant *gyrA* and WT *penA* alleles, including one isolate with a mutant (S87I) *parC* allele, 4/26 (2012 to 2014) possessed WT *gyrA* and mutant *penA* alleles, and 1/26 (2013) possessed mutant alleles for both *gyrA* and *penA*. All three Chinese MenC carrier isolates (2016 to 2017) possessed mutant alleles for both *gyrA* and *penA*. Among the Chinese invasive MenB lineage 2c isolates, 4/9 possessed WT *gyrA* and *penA* alleles, 1/9 possessed WT *gyrA* and mutant *penA* alleles, 2/9 possessed mutant *gyrA* and WT *penA* alleles, and 2/9 possessed mutant alleles for both *gyrA* and *penA*. The single Chinese invasive MenC isolate possessed mutant *gyrA* and WT *penA* alleles. A further MenB isolate with unknown disease status possessed WT *gyrA* and *penA* alleles.

All 17 lineage 2c RoW isolates possessed WT *gyrA* alleles and mutant *penA* alleles.

#### Diffuse isolates

Among the diffuse carrier isolates 2/2 MenB (2009 and 2011) and 3/8 MenC isolates (1973 to 1978) possessed WT *gyrA* and *penA* alleles. A further 4/8 MenC isolates (2005 to 2008) possessed mutant *gyrA* and WT *penA* alleles, while 1/8 MenC isolates (1980) possessed WT *gyrA* and incomplete *penA* alleles.

Among the diffuse invasive isolates, 2/3 MenB isolates (1985 and 2011) possessed WT alleles for both *gyrA* and *penA*. The remaining isolate (2009) possessed mutant *gyrA* and *penA*.

The two diffuse isolates with unknown disease status (1980 and 1985) possessed WT *gyrA* and *penA* alleles.

### Subcapsular vaccine antigen genes

**Fig 4:**
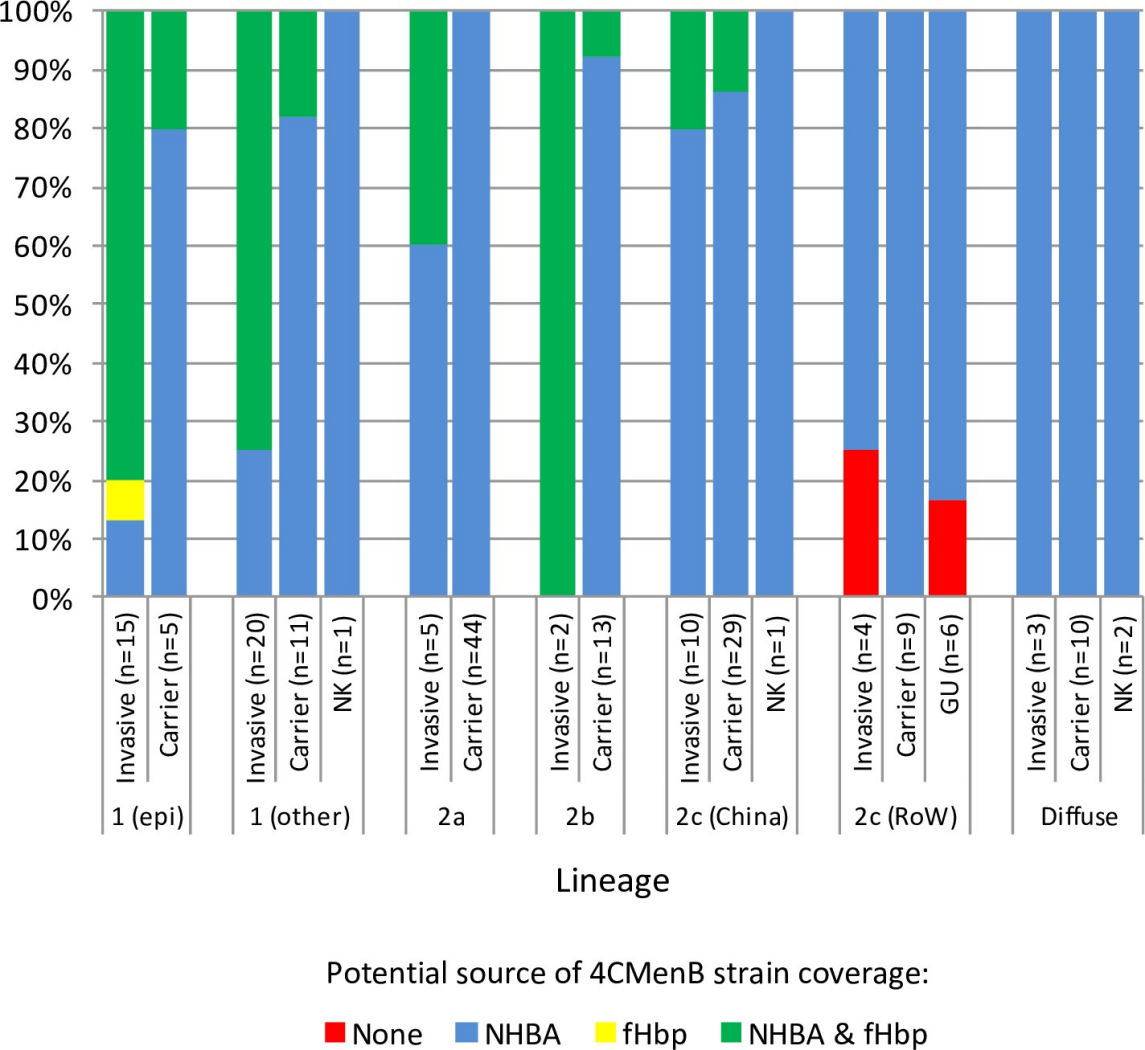
Genotypic assessment of potential antigen-specific strain coverage of cc4821 lineages/sublineages by 4CMenB. Potential strain coverage was estimated based on the most conservative criteria that any NHBA peptide and any fHbp variant 1 peptide is potentially covered.

None of the cc4821/ST-14840 isolates possessed alleles for PorA P1.4 or NadA. Two isolates had incomplete *nhba* sequences and one, a UK invasive rectal swab isolate from 2017, had a frameshifted *nhba* allele. The remaining 185 isolates possessed alleles for one of 32 intact NHBA peptides, 22 of which were unique to China, and 27 of which were only, or predominantly, observed in cc4821 on the PubMLST *Neisseria* database (accessed 24/06/20). 4CMenB strain coverage of all but one of the encoded NHBA peptides was unpredictable according to gMATS. The remaining peptide, peptide 10 was predicted by gMATS to be covered and occurred in a single lineage 2a MenB carrier isolate from 2013 (this isolate possessed an allele for a non-covered variant 2 fHbp peptide). The majority of isolates 144/188 possessed fHbp variant 2 or 3 alleles and a further two isolates, a Chinese invasive MenC lineage 1 (non-epidemic clone) isolate from 2011 and a Chinese lineage 2c MenC carrier isolate from 2017, possessed a truncated allele (nonsense mutation) and a complete deletion of *fhbp*, respectively. The remaining 42 isolates possessed alleles for one of 13 fHbp variant 1 peptides, eight of which were only, or predominantly, found in cc4821, and six of which were unique to China on the PubMLST *Neisseria* database (accessed 24/06/20). All 13 peptides afforded unpredictable 4CMenB strain coverage according to gMATS. Given the general absence of gMATS-predictable strain coverage, potential strain coverage was estimated based on the most conservative criteria that any NHBA peptide and any fHbp variant 1 peptide is potentially covered. Potential antigen-specific strain coverage of cc4821 lineages/sublineages by 4CMenB is shown in **Fig 4**. A detailed breakdown of lineage-specific potential 4CMenb strain coverage is as follows:

#### Lineage 1

At least 50 of the 52 lineage 1 isolates were potentially covered by NHBA though this was heavily reliant on peptide 503 (n = 43) being covered. The two remaining isolates had incomplete *nhba* sequences. Interestingly, only 3/16 carrier isolates, including 1/5 epidemic clone carrier isolates (1/4 MenB and 2/10 MenC) were also potentially covered by fHbp. By contrast, 28/35 of the invasive isolates (3/6 MenB and 25/29 MenC), including 13/15 invasive epidemic clone isolates, were potentially covered by fHbp. One invasive MenC (2011) isolate possessed a truncated *fhbp* allele with a nonsense mutation.

#### Lineage 2a

All 49 lineage 2a isolates were potentially covered by NHBA though this was heavily reliant on peptides 910 and 688 being covered since these accounted for a half and a quarter of the isolates, respectively. Among the invasive isolates, 2/5 were also potentially covered by virtue of one of two different fHbp peptides.

#### Lineage 2b

All of the lineage 2b isolates were potentially covered by NHBA, though this was reliant on peptide 697 being covered. Additionally, 1/13 carrier isolates (MenW, 2014) and the two invasive MenC isolates were potentially covered by virtue of fHbp.

#### Lineage 2c

All of the 40 Chinese lineage 2c isolates were potentially covered by NHBA though this was mainly reliant on coverage of peptides 669 (n = 22) and 697 (n = 9). In addition, 4/25 carrier isolates and 2/10 invasive isolates (MenB, 2011 and MenC, 2005) were potentially covered by fHbp. An *fhbp* gene was lacking in one MenC carrier isolate (2017) and was replaced by *N*. *lactamica* sequence as previously reported among proposed ST-286 complex isolates [30].

Among the lineage 2c RoW isolates, 16/17 were potentially covered by virtue of NHBA, in particular peptide 669 (n = 15). One MenB isolate from a rectal swab from an invasive disease patient possessed a frameshifted *nhba* allele. None of the RoW isolates were covered by fHbp.

#### Diffuse isolates

Potential 4CMenB coverage of the 15 diffuse isolates was limited to NHBA, in particular peptide 669 (n = 10).

### Distribution of the nitrite reductase (*aniA*) gene previously associated with adaptation to the anogenital niche

The acquisition of a working nitrite reductase (*aniA*) gene has previously been implicated in the adaptation of meningococci to the anogenital niche [17, 18]. The distribution of *aniA* alleles within the cc4821 population structure is shown in **S6 Fig**. A number of RoW cluster and lineage 2c isolates possessed *aniA* (NEIS1549) allele 8 that was unique to these and a small number of other ST-3200 (cc4821) related isolates on the PubMLST *Neisseria* database (accessed 21/07/20). A genome comparison of all lineage 2c isolates in terms of all NEIS loci showed that isolates with *aniA* allele 8 also possessed a unique nitric oxide reductase (*norB*; NEIS1548) allele–allele 379. Most other lineage 2c isolates possessed *aniA* allele 287 and *norB* allele 838 in common with diverse isolates from lineages 2a and 2b. The most commonly observed alleles of the loci immediately upstream and downstream of *aniA* and *norB* did not differ between the two sets of isolates suggesting a possible recombination event. A nucleotide alignment of *aniA*, *norB* and flanking genes between isolates representative of the putative ancestral (*aniA* allele 287 and *norB* allele 838) and putative recombinant (*aniA* allele 8 and norB allele 379) states identified a potentially horizontally acquired region of 2022 bp covering 82% of the 5’ end of *aniA*, 30% of the 5’end of *norB*, and the 381 bp intergenic region **(S7 Fig)**. The extent of the change in the respective peptides was relatively modest comprising P23Q, A43T, T45A and the addition of A27, P28, A29 and E30 for AniA **(S8A Fig)**, and I133V, V146A, D206G, T208V for NorB **(S8B Fig)**.

A BLAST search of all genomes (>2Mb) on the PubMLST *Neisseria* database (n = 30,044; accessed 1^st^ September 2020) for the putatively acquired 2022 bp sequence identified n = 103 exact, full-length matches, all of which were meningococcal. Thirty eight of these belonged to, or were closely related to, cc4821, including n = 29 cc4821 lineage 2c isolates from the current study with *aniA* allele 8 (n = 28) or *aniA* allele 733 (n = 1); five cc4821 carriage study isolates from the UK and Sweden that were not included in the current study (all *aniA* allele 8); and four clonal complex-unassigned isolates with <4 MLST alleles in common with ST-4821 (all *aniA* allele 8). The latter four isolates each had 4 to 5 loci in common with ST-3200. The cc8 isolate descended of cc4821 (*aniA* allele 8) was also identified. The remaining BLAST hits were for MenA ST-4 complex (n = 14; 1915 to 1972) and MenA ST-5 complex (n = 48; 1966 to 2014) isolates and two isolates with incomplete MLST profiles but matching cc5 at six loci.

### *mtrC* analysis among anogenital isolates

The six lineage 2c RoW anogenital isolates possessed *mtrC* (NEIS1634) allele 93 encoding an intact peptide.

## Discussion

High-resolution phylogenetic analyses have revolutionised our understanding of meningococcal population structures and our ability to identify and track existing and emerging strains and outbreaks on a global level [31, 32]. Such analyses benefit from the inclusion of geo-temporally diverse isolates to provide the broadest possible view of the corresponding diversity and to put the constituent strains in context. To this end, our study included all currently available cc4821 genomes on the PubMLST database (accessed 12/09/19).

A key finding of our study is the existence of four well defined lineages, versus the two that were previously identified [5, 6]. Lineage 1 corresponds to the previously identified group I/1 which includes the predominantly MenC epidemic clone with PorA P1.7–2,14 [6]. Lineages 2a, 2b, and 2c, and a number of diffuse isolates/singletons, collectively correspond to the previously designated group II/2. It was previously noted that group 2 was relatively diverse but the inclusion of more than five times as many genomes in the present study has helped to resolve the constituent lineages. The star-like arrangement of the four lineages along with the non-cc4821 outgroup on the phylogeny suggests that the four lineages have diverged concurrently from an early common ancestor, however, the relative closeness of lineages 2a, 2b and 2c to one-another and the benefits of maintaining continuity with previous publications justifies the chosen lineage designations (2a, 2b and 2c).

Interestingly, with the exception of a lineage 1 epidemic clone MenC case isolate from Japan in 2017, all of the remaining 17 non-Chinese isolates, from nine countries on three continents (2007 to 2018), exclusively belonged to a distinct cluster (the RoW cluster) within lineage 2c. Furthermore, whilst PorA subtype P1.17–6,23 and closely-related subtypes were only associated with the RoW cluster among our genome panel, they also accounted for 14/27 PorA subtypes among non-Chinese, non-genomic isolate submissions on the PubMLST *Neisseria* database (accessed 13^th^ July 2020; S2 Table). The lineage-specific distribution of several predominant Sequence Types suggests that the previously reported ‘ST-3200 group’ of invasive MenB isolates in Taiwan from 1996 to 2002 [33] would also form part of the broader lineage 2c. The global expansion of this particular sublineage may be due to an unknown feature of the strain making it more transmissible in these populations or may simply be opportune e.g. due to an international mass gathering [34].

More than a third (6/17) of the lineage 2c RoW cluster isolates were from an anogenital site among MSM. Although two groups of isolates (a group of three and a group of two) were from patients attending sexual health services that utilised common microbiology laboratories (at opposite ends of the UK), the corresponding isolates were distributed over time (2011, 2012 and 2013; and 2017 and 2018, respectively) and within the population structure of the corresponding cluster. Furthermore, the second group also differed from one-another by serogroup. This, along with the finding of the recent msm carriage study in New York (USA) [14], may be indicative of a propensity for this cluster to infect the anogenital site as has previously been observed with ST-11 complex lineage 11.2 strains [15, 16, 18]. One factor in the emergence of anogenital-associated cc11 strains was the acquisition of an active nitrite reductase (*aniA*) gene [15, 18]. Among the cc4821 lineages, only lineage 1 lacked *aniA* functionality, however, 14/17 lineage 2c RoW isolates, including the anogenital isolates, and 15/40 Chinese lineage 2c isolates shared a putative ancestral recombination involving partial sequences of the divergent *aniA* and *norB* genes along with the intergenic/promoter regions. Although the corresponding sequence was exclusively meningococcal on the PubMLST database, its initial origin was likely in MenA-associated lineages dating back to 1915. It is possible that the newly generated alleles or the newly acquired intergenic region (containing promoter regions) where many of the changes occurred, are more efficient than the existing ones. This, however, requires further investigation. Formal bioinformatic analyses are also required to confirm the origin of the putatively horizontally acquired sequence. Another possible explanation for this particular cluster alone being observed in anogenital sites is that Chinese anogenital meningococci from other lineage 2c clusters or the other cc4821 lineages may simply not be collected, sequenced, posted or identified as such on PubMLST. Interestingly, lineage 2c also included a rare isolate lacking an *fhbp* gene and therefore unable to express fHbp which has also previously been implicated in adaptation to the anogenital niche [18]. None of the anogenital isolates possessed LOF mutations in *mtrC* that have been associated with cervical infection in gonococci and urogenitally adapted meningococci from uretheritis outbreaks among heterosexual males [19]. One possible explanation for this is that cc4821 isolates were all isolated from MSM and the cervix may not have been encountered in recent ancestral isolates.

The absence of the lineage 1 epidemic clone outside of Asia is surprising given that it has caused most cases of cc4821 disease within China. This may, in part, be due to a lack of corresponding genomes. Canada, for example, has reported IMD due to a P1.7–2,14:ST-4821 isolate [31, 35] but no genome is currently available. On our phylogeny, with the exception of a single diffuse isolate from China in 1980, PorA P1.7–2,14 was only observed within the discrete cluster representing the epidemic clone. This supports PorA P1.7–2,14 as a specific marker for the clone among cc4821 isolates. In addition, PorA VR1 P1.7–2 alone was a strong marker for the epidemic clone cluster accounting for 22/23 (96%) isolates. Three of the epidemic clone isolates possessed a different VR2. In light of this, the lack of the epidemic clone outside of China is further supported by available PorA data among non-Chinese, non-genomic, cc4821 isolate submissions on the PubMLST *Neisseria* database (accessed 13^th^ July 2020). None of the isolates from Europe (France, n = 2, 2009 and 2011; Spain, n = 2, 2018; Czech Republic, n = 1, 2007; Italy, n = 1, 2020), North America (USA, n = 14, 2015 to 2019), South America (Brazil, n = 1, 2014), Australasia (Australia, n = 2, 2012 and 2014; New Zealand, n = 1, 2017) or Asia (Taiwan, n = 2, 2004 and 2005; Vietnam, n = 1, 2012) possessed the epidemic clone-associated PorA (P1.7–2,14) (S2 Table).

The widespread distribution of fluoroquinolone resistance in cc4821 is well documented [36]. The present study confirms the distribution of diverse resistance-associated (MICs > 0.03 mg/L) mutant *gyrA* alleles throughout the four cc4821 lineages and, with the exception of lineage 1, these were interspersed with isolates possessing susceptibility-associated (MICs ≤0.03 mg/L) WT alleles. A small number of well distributed isolates also possessed mutant *parC* alleles that may further increase fluoroquinolone MICs. Notably, the RoW cluster does not possess a mutant *gyrA*, however, it may be susceptible to stable acquisition of fluoroquinolone resistance given the situation in the broader clonal complex and the gradual emergence of fluoroquinolone resistance in Western countries [35, 37–39]. Interestingly, all of the RoW isolates possessed a mutant *penA* allele associated with reduced penicillin susceptibility (0.094 to 1 mg/L). Although diverse mutant *penA* alleles were found among Chinese isolates in each of the four lineages, they were relatively rare. As the present study constituted a genomic survey, we focussed on antibiotic resistance/reduced susceptibility determinants commonly associated with cc4821, rather than phenotypic MICs. Further work may include consideration of other potential resistance determinants such as multidrug efflux systems or beta lactamase, the latter of which has recently been reported among meningococci in North America and Europe [37, 40, 41].

Potential 4CMenB strain coverage of lineages 2a, 2b and 2c, including the RoW cluster was heavily reliant on NHBA. As the corresponding peptides are rare among existing MATS datasets there is a critical need for MATS analysis of isolates representing each of the main peptides. This may then be used to extend the current list of covered, not covered, and unpredictable isolates as defined by gMATS [12]. Perhaps reassuringly, more than half of the lineage 1 isolates, including 85% (28/33) of invasive isolates were also potentially covered by fHbp. This vaccine may, therefore, be an option if an fHbp lineage 1-possessing MenB strain should emerge to cause a large outbreak or epidemic. The majority of isolates without a variant 1 *fhbp* possessed a variant 2 or 3 gene and so the majority of isolates are potentially covered by Men-fHbp, though this would need confirming with the MeASurE assay [11]. Of concern, two isolates lacked a functional *fhbp* and one isolate lacked a function *nhba*. Such isolates and their corresponding strains have infrequently been reported before [30, 34, 42] and should be monitored due to the potential for escape of subcapsular vaccines. They should also be considered when devising new vaccine formulations.

In conclusion, cc4821 can now be considered in terms of four distinct lineages and the phylogeny herein can form a template with which to analyse genomes from prevailing cases. A small and distinct cluster of lineage 2c is responsible for the majority of cases outside of China and may have an affinity for anogenital sites. In light of emerging fluoroquinolone and penicillin resistance, uncertain strain coverage by subcapsular vaccines, and the repeated emergence of MenB cc4821 strains through capsule switching, there is an urgent need to test isolates representative of each lineage in the MATS and MeASurE assays.

## Supporting information

S1 Fig Distribution of previously designated group I/1 and group II/2 isolates within cc4821 population structure. (DOCX)

S2 Fig Distribution of Chinese provinces represented within the cc4821 population structure. (DOCX)

S3 Fig Distribution of genotypic PorA subtypes in cc4821 lineage 1. (DOCX)

S4 Fig Distribution of sequence types within cc4821 population structure. (DOCX)

S5 Fig Distribution of fluoroquinolone resistance-associated *gyrA* and *parC* alleles and *penA* alleles associated with reduced penicillin susceptibility within the cc4821 population structure. (DOCX)

S6 Fig Distribution of nitrite reductase (*aniA*) alleles within the cc4821 population structure. (DOCX)

S7 Fig Alignment of *aniA*, *norB* and flanking genes between cc4821 lineage 2c isolates representative of the ancestral and novel recombinant states. (DOCX)

S8 Fig Alignment of AniA and NorB from putative ancestral and recombinant cc4821 lineage 2 isolates. (DOCX)

S1 Table Isolate/genome listing, genotypic data and accession numbers. (XLSX)

S2 Table PorA subtypes among non-Chinese, non-genomic PubMLST submissions highlighting P1.17–6,23 and closely-related subtypes that were only associated with the RoW cluster among the genome panel. (DOCX)
