# Supplementary material for: Genomic analysis of the meningococcal ST-4821 complex–Western clade, potential sexual transmission and predicted antibiotic susceptibility and vaccine coverage
Source: PLoS One. 2020 Dec 10;15(12):e0243426. doi: 10.1371/journal.pone.0243426 (PMC7728179; doi:10.1371/journal.pone.0243426)
Supplement: S1 Fig — (DOCX) [file pone.0243426.s001.docx]

**S1 Fig.** Distribution of previously designated group I/1 and group II/2 isolates within cc4821 population structure.

The cc4821 isolates formed four main sublineages (sublineages 1, 2a, 2b and 2c) interspersed with more diffuse isolates. Lineage 1 included previously designated group I/1 and epidemic clone isolates. Lineages 2a, 2b and 2c included diverse group II/2 isolates. The phylogeny was based on a core genome (1605 loci) comparison. The scale bar represents the number of different loci.
