# Supplementary material for: Genomic analysis of the meningococcal ST-4821 complex–Western clade, potential sexual transmission and predicted antibiotic susceptibility and vaccine coverage
Source: PLoS One. 2020 Dec 10;15(12):e0243426. doi: 10.1371/journal.pone.0243426 (PMC7728179; doi:10.1371/journal.pone.0243426)
Supplement: S2 Fig — (DOCX) [file pone.0243426.s002.docx]

**S2 Fig.** Distribution of Chinese provinces represented within the cc4821 population structure.

Each of the main sublineages represented multiple Chinese provinces. The phylogeny was based on a core genome (1605 loci) comparison. The scale bar represents the number of different loci.
