# Supplementary material for: Genomic analysis of the meningococcal ST-4821 complex–Western clade, potential sexual transmission and predicted antibiotic susceptibility and vaccine coverage
Source: PLoS One. 2020 Dec 10;15(12):e0243426. doi: 10.1371/journal.pone.0243426 (PMC7728179; doi:10.1371/journal.pone.0243426)
Supplement: S3 Fig — (DOCX) [file pone.0243426.s003.docx]

**S3 Fig.** Distribution of PorA subtypes in cc4821 lineage 1.

Lineage 1 included diverse PorA subtypes. PorA P1.7-2,14 was observed within a discreet cluster representing the epidemic clone. Three isolates clustering with the epidemic clone isolates possessed a different PorA subtype – P1.21-2,23-6, P1.7-2,9, and P1.7-2,14-31. Other PorA subtypes observed once among the lineage 1 isolates were P1.18,25-11, P1.20,23, P1.20,23-2, P1.20,23-3, and P1.20,23-9. The phylogeny was based on a core genome (1605 loci) comparison. The scale bar represents the number of different loci.
