# Supplementary material for: Genomic analysis of the meningococcal ST-4821 complex–Western clade, potential sexual transmission and predicted antibiotic susceptibility and vaccine coverage
Source: PLoS One. 2020 Dec 10;15(12):e0243426. doi: 10.1371/journal.pone.0243426 (PMC7728179; doi:10.1371/journal.pone.0243426)
Supplement: S4 Fig — (DOCX) [file pone.0243426.s004.docx]

**S4 Fig.** Distribution of Sequence Types within cc4821 population structure.

ST-4821 was distributed between lineage 1 and several diffuse isolates. ST-5664 and ST-9454 were confined to lineage 2a. ST-8491 was confined to MenW isolates of lineage 2b. ST-3200 and ST-5798 were confined to lineage 2c. The phylogeny was based on a core genome (1605 loci) comparison. The scale bar represents the number of different loci.
