# Supplementary material for: Genomic analysis of the meningococcal ST-4821 complex–Western clade, potential sexual transmission and predicted antibiotic susceptibility and vaccine coverage
Source: PLoS One. 2020 Dec 10;15(12):e0243426. doi: 10.1371/journal.pone.0243426 (PMC7728179; doi:10.1371/journal.pone.0243426)
Supplement: S5 Fig — (DOCX) [file pone.0243426.s005.docx]

**S5 Fig.** Distribution of fluoroquinolone resistance-associated *gyrA* and *parC* alleles and *penA* alleles associated with reduced penicillin susceptibility within the cc4821 population structure.

Resistance/susceptibility-associated *gyrA* and *penA* alleles were broadly dispersed within lineage 2a, 2b and 2c isolates. Lineage 1 isolates mostly possessed resistance-associated *gyrA* and susceptibility-associated *penA* alleles whilst the Rest of the World cluster (RoW) possessed susceptibility-associated *gyrA* alleles and *penA* alleles associated with reduced susceptibility. A small number of isolates in lineages 1, 2a and 2c also possessed mutant *parC* alleles. The phylogeny was based on a core genome (1605 loci) comparison. The scale bar represents the number of different loci. *gyrA* alleles were considered to be associated with fluoroquinolone resistance (MIC > 0.03 mg/L) if they possessed mutations affecting residues T91 or D95, with or without *parC* mutations (D86, S87, S88 or E91), of the corresponding peptides. *penA* alleles were considered to be associated with reduced penicillin susceptibility (0.094 to 1 mg/L) if they encoded the following mutations: F504L, A510V, I515V, H541N and I566V.
