# Supplementary material for: Genomic analysis of the meningococcal ST-4821 complex–Western clade, potential sexual transmission and predicted antibiotic susceptibility and vaccine coverage
Source: PLoS One. 2020 Dec 10;15(12):e0243426. doi: 10.1371/journal.pone.0243426 (PMC7728179; doi:10.1371/journal.pone.0243426)
Supplement: S6 Fig — (DOCX) [file pone.0243426.s006.docx]

**S6 Fig.** Distribution of nitrite reductase (*aniA*) alleles within the cc4821 population structure.

Lineage 1 isolates lacked the *aniA* gene. The remaining isolates possessed diverse *aniA* alleles. The presumed ancestral allele was allele 287, on the basis that it was observed throughout lineages 2a, 2b and 2c. A large lineage 2a cluster, including the Rest of the World (RoW) cluster possessed a putative recombinant allele, allele 8. The phylogeny was based on a core genome (1605 loci) comparison. The scale bar represents the number of different loci. fs = frameshifted allele. *Isolate does not have a factor H-binding protein (*fhbp)* gene.
