# Supplementary material for: Genomic analysis of the meningococcal ST-4821 complex–Western clade, potential sexual transmission and predicted antibiotic susceptibility and vaccine coverage
Source: PLoS One. 2020 Dec 10;15(12):e0243426. doi: 10.1371/journal.pone.0243426 (PMC7728179; doi:10.1371/journal.pone.0243426)
Supplement: S7 Fig — (DOCX) [file pone.0243426.s007.docx]

**S7 Fig.** Alignment of *aniA*, *norB* and flanking genes between cc4821 lineage 2c isolates representative of the putative ancestral (92580_310827) and novel (92575_131259) recombinant states.

Identical bases are denoted by dots/highlighted black. All differences occur towards the centre of the alignment in the 5’ end of the divergent *aniA* and *norB* genes and the intergenic region. Dashes represent gaps.
