# Supplementary material for: Genomic analysis of the meningococcal ST-4821 complex–Western clade, potential sexual transmission and predicted antibiotic susceptibility and vaccine coverage
Source: PLoS One. 2020 Dec 10;15(12):e0243426. doi: 10.1371/journal.pone.0243426 (PMC7728179; doi:10.1371/journal.pone.0243426)
Supplement: S8 Fig — (DOCX) [file pone.0243426.s008.docx]

**S8 Fig.** Alignment of AniA and NorB from putative ancestral and recombinant cc4821 lineage 2 isolates.

Identical bases are denoted by dots/highlighted black. Similar amino acids are highlighted grey.
