# Supplementary material for: Genomic analysis of the meningococcal ST-4821 complex–Western clade, potential sexual transmission and predicted antibiotic susceptibility and vaccine coverage
Source: PLoS One. 2020 Dec 10;15(12):e0243426. doi: 10.1371/journal.pone.0243426 (PMC7728179; doi:10.1371/journal.pone.0243426)
Supplement: S2 Table — (DOCX) [file pone.0243426.s010.docx]

**S2 Table.** PorA subtypes among non-Chinese, non-genomic PubMLST submissions highlighting P1.17-6,23 and closely-related subtypes that were only associated with the RoW cluster among the genome panel.

| **id** | **isolate** | **country** | **year** | **PorA_VR1** | **PorA_VR2** |
| --- | --- | --- | --- | --- | --- |
| 84631 | NZ18MI0003 | New Zealand | 2017 | 5 | 2 |
| 6772 | N93-03001 | Taiwan | 2004 | 5 | 2-49 |
| 53740 | M41275 | USA | 2016 | 17 | 9 |
| 9734 | NM02740 | USA | 2007 | 20 | 23 |
| 70915 | M50656 | USA | 2019 | 20 | 23 |
| 47222 | M38915 | USA | 2015 | 20 | 23-2 |
| 8134 | 0068/07 | Czech Republic | 2007 | 20 | 23-7 |
| 64026 | 23218 | Spain | 2018 | 20 | 23-14 |
| 19785 | LNP26073 | France | 2011 | 22 | 14 |
| 7940 | N9403011-C | Taiwan | 2005 | 12-14 | 13-20 |
| 72728 | 1292 | Vietnam | 2012 | 5-1 | 2-2 |
| 70931 | M51537 | USA | 2019 | 22-1 | 30 |
| 35857 | 14M10273 | Australia | 2014 | 17-6 | 23 |
| 14245 | 25241 | France | 2009 | 17-6 | 23 |
| 72688 | 3028 | Italy | 2020 | 17-6 | 23 |
| 64025 | 23169 | Spain | 2018 | 17-6 | 23 |
| 49265 | M37056 | USA | 2015 | 17-6 | 23 |
| 53749 | M41304 | USA | 2016 | 17-6 | 23 |
| 61436 | M44733 | USA | 2017 | 17-6 | 23 |
| 70918 | M50812 | USA | 2019 | 17-6 | 23 |
| 91709 | M47524 | USA | 2018 | 17-6 | 23 |
| 91716 | M49929 | USA | 2018 | 17-6 | 23 |
| 53730 | M41240 | USA | 2016 | 17-6 | 23-2 |
| 38174 | M37121 | Brazil | 2014 | 17-6 | 23-6 |
| 70881 | M48572 | USA | 2018 | 17-6 | 23-6 |
| 59260 | M45920 | USA | NK | 17-6 | NK |
| 35845 | 12M0320 | Australia | 2012 | 22-15 | 4 |
